# Burden of diarrhoeal diseases among hospitalised patients in Thailand: a retrospective national database analysis (2014–2022)

**DOI:** 10.1016/j.lansea.2026.100761

**Published:** 2026-04-06

**Authors:** Suppasit Srisaeng, Sakkarin Chirapongsathorn, Ngamphol Soonthornworasiri, Kittiyod Poovorawan, Wirichada Pan-Ngum

**Affiliations:** a Department of Tropical Hygiene, Faculty of Tropical Medicine, Mahidol University, Bangkok, Thailand; b Office of Disease Prevention and Control Region 6, Chonburi, Ministry of Public Health, Nonthaburi, Thailand; c Division of Gastroenterology and Hepatology, Department of Medicine, Phramongkutklao Hospital College of Medicine Royal Thai Army, Bangkok, Thailand; d Department of Clinical Tropical Medicine, Faculty of Tropical Medicine, Mahidol University, Bangkok, Thailand; e Mahidol-Oxford Tropical Medicine Research Unit (MORU), Faculty of Tropical Medicine, Mahidol University, Bangkok, Thailand

**Keywords:** Diarrhoea, Thailand, Rotavirus, *Salmonella*, *Clostridioides difficile*, Costs

## Abstract

**Background:**

Acute diarrhoea remains one of the leading causes of morbidity and mortality worldwide. Most existing research has focused on children younger than 5 years of age, while data from Thailand are limited. In this study we characterised trend of diarrhoeal admissions, associated Disability-adjusted life years (DALYs) and determined predictors of mortality and high-cost admissions in Thailand.

**Methods:**

We analysed Thailand's National Health Security Office inpatient claims database for 2014–2022. We estimated admission rates (ASRs), costs of admission ($PPP), and DALYs for pre-pandemic (2014–2019) and pandemic (2020–2022) periods. Predictors of mortality and high-cost admission were assessed using logistic regression. Rotavirus vaccine impact was evaluated among children younger than 5 years of age.

**Findings:**

Among 3,041,699 total admissions, 54·3% were female, median age was 22 years (IQR 3–61). ASR fell from 686·5/100,000 pre-pandemic to 474·5/100,000 pandemic, while mean cost rose from $PPP 372 to 539. The case fatality ratio was 0·6 (18,782 deaths). Total burden was 1,124,618 DALYs; mean annual DALYs fell from 132,366 to 110,140. Children younger than 5 years of age accounted for 31·4% of admissions, whereas adults older than or equal to 60 years of age accounted for 25·7% of admissions and 64·6% of total deaths. Older age, male sex, referral hospitals, central region, stroke, sepsis, respiratory failure, and *Salmonella* or *Clostridioides difficile* infection was associated with mortality or high-cost admission. Each 1% increase in rotavirus vaccine coverage was associated with 2·0% reduced rotavirus admissions.

**Interpretation:**

Thailand's diarrhoeal admissions were concentrated among young children and older adults, with mortality and high-cost admissions driven by *Salmonella* and *C. difficile infections*. Improved water, sanitation, rotavirus vaccination, and hospital preparedness are essential to tackle the diarrhoeal disease burden.

**Funding:**

This research was funded by 10.13039/100004440Wellcome (grant number 315982/Z/24/1). For the purposes of open access, the authors have applied a CC BY public copyright license to any Author Accepted Manuscript version arising from this submission.

**Research in context**

**Evidence before this study**

We searched PubMed, Google Scholar, MEDLINE, Web of Science, and the Thai-Journal Citation Index for articles published from database up to October 31, 2025, without language restrictions. We used search terms including (“diarrhea” OR “gastroenteritis”) AND (“Thailand”) AND (“burden” OR “mortality” OR “cost” OR “economic”). We prioritized nationwide studies using population-level datasets reporting hospital admissions, mortality, cost, or DALYs across ages. Comprehensive estimates for the general population rely on data from 2010. We found no recent nationwide studies analyzing diarrheal burden in Thai adults or the elderly. Recent adult data are limited to single-center or regional studies, which carry a risk of selection bias and fail to capture the national economic impact or the epidemiological shifts occurring during the COVID-19 pandemic.

**Added value of this study**

This study provides the largest nationwide analysis of diarrhea-related hospital admissions in Thailand, covering 3·12 million admissions from 2014 to 2022, describing temporal trends, costs, DALYs, and factors associated with in-hospital mortality and high-cost admissions. This study bridges the evidence gap between pediatric and adult epidemiology by defining a “U-shaped” burden: while admission volume remains highest in young children, mortality has shifted decisively to the geriatric population. We provide the national-level quantification of the COVID-19 pandemic's impact on enteric disease, revealing that a reduction in admission volume was paradoxically accompanied by increased case severity and costs. Crucially, we provide the national-level assessment of the rotavirus vaccine introduced in 2020, establishing a significant dose–response relationship where every 1% increase in vaccine coverage correlates with a 2·0% reduction in rotavirus-related admissions. Furthermore, we identify *Salmonella* and *Clostridioides difficile* as the specific pathogens driving high-cost care and mortality in the elderly, providing new evidence to guide resource allocation.

**Implications of all the available evidence**

The combined evidence indicates that Thailand has successfully reduced the burden of traditional water-borne pathogens and is making significant progress against pediatric rotavirus through vaccination. Strengthening water and sanitation systems, sustaining high rotavirus vaccination coverage, and improving diagnostic capacity and hospital preparedness for severe complications are essential. The public health challenge has now transitioned to geriatric enteric sepsis. Clinical guidelines and health policy must shift focus toward the early management of systemic complications in elderly patients with comorbidities. The rising economic burden and severity of *C. difficile* infections necessitate the urgent implementation of rigorous antimicrobial stewardship programs and updated reimbursement models that account for the increasing complexity of care in an aging society.

## Introduction

Acute diarrhoea is defined as the passage of three or more loose stools in a 24-h period.^1^ Viral diarrhoea is more common in cooler, drier seasons, while bacterial diarrhoea peaks in warmer, rainy seasons.^2^ Key pathogens include rotavirus, *Shigella*, norovirus and adenovirus, with rotavirus causing 33·3% of diarrhoea-related admissions in children under five.^3^^,^^4^

Acute diarrhoea is a major global public health concern globally. Each year, it is estimated to cause 67·3 million adult and 5·8 million child cases, 1·17 million deaths, with a mortality rate of 51·72 per 100,000, and contributes to 59·0 million disability-adjusted life years (DALYs) globally.^5^ Although mortality due to diarrhoea has decreased in high-income countries, it contributes towards a high burden in low- and middle-income countries due to limited access to quality water, sanitation and hygiene (WASH) practices.^6^

In Thailand, a 2010 nationwide analysis reported 214,722 adult hospital admissions for acute diarrhoea, with a 0·5% case fatality ratio (CFR).^7^ Subsequent data from 2015 to 2019 showed an average inpatient (IPD) admission rate of 33·8 per 1000 among children younger than 5 years of age and mortality rates of 0·71–1·16 per 100,000.^8^ In 2023, Thailand's digital disease surveillance recorded 677,611 total outpatient and inpatient patients, among which 128,781 (19%) were children younger than five years of age.^9^

Strategies to mitigate this burden among children in Thailand have focussed on implementation of Rotavirus vaccination. Following the 2020 nationwide introduction of the rotavirus vaccine as part of the Expanded Program on Immunization (EPI), outpatient visits due to rotavirus decreased by 17·8%.^4^ Diarrhoeal illness also creates a substantial economic burden in Thailand. A 2014 study estimated a societal cost of 10,165 Thai baht (THB) per hospitalised case, approximately USD 822·68 purchasing power parity ($PPP).^10^

Though few recent studies have analysed trends in admission costs and DALYs for acute diarrhoea, evidence on the effects of length of stay (LOS), medical interventions, and COVID-19 policies on diarrhoeal admission costs and mortality is limited, as are trend analysis. Addressing these gaps is essential for guiding future public health interventions and policy. Therefore, in this study we aimed to characterise diarrhoeal admissions, costs, and DALYs; describe pathogen trends; and identify predictors of mortality and high-cost admissions. Furthermore, we sought to evaluate the impact of the COVID-19 pandemic, on total admissions and per-case severity. We have also performed a subgroup analysis among children younger than 5 years of age to understand the impact of rotavirus vaccination on hospitalisations.

## Methods

We conducted a retrospective observational study using routinely collected administrative inpatient claims data from Thailand's National Health Security Office (NHSO), between the years 2014 and 2022. We performed time-stratified descriptive and analytical comparisons between the pre-pandemic period (2014–2019) and the COVID-19 pandemic period (2020–2022) and evaluated the impact of rotavirus vaccine introduction in 2020. The dataset comprised all ages inpatient admission records from both public and private hospitals with a primary diagnosis of acute diarrhoea or related gastrointestinal conditions. The data only included admissions of individuals belonging to Thai nationality. The unit of analysis was the hospital admission. Data were sourced from major insurance schemes: the Universal Coverage Scheme (UCS) and the Civil Servant Medical Benefits Scheme (CSMBS; this scheme covers government officers). Rotavirus vaccine coverage data were obtained from the Ministry of Public Health open data portal.^11^ Mortality was ascertained using NHSO discharge status and linkage to Thailand's death registry.

Included admissions were based on primary diagnosis, using ICD-10 codes encompassing intestinal infectious diseases (A00–A09), parasite infestations (B77–B79), other specified intestinal infections (B81–B82), bacterial and viral agents (B95–B97), other noninfective gastroenteritis and colitis (K52), enterovirus infection (B34·1) and excluding non-intestinal origin (A02·8, A06·4–A06·9, B77·8, B78·1, B78·7 and K52·1).

Records were excluded if they were missing age or sex, were duplicates of a same admission day, or were inter-hospital transfers. Diseases that mimic diarrhoea, such as malignant neoplasm of the colon (primary diagnosis C18), were also excluded. Records with a date of death difference of more than 31 days between the NHSO data and Thailand's deaths registry were excluded to ensure mortality was temporally related to the index admission.

Acute diarrhoea was defined according to the ICD-10 inclusion criteria. Non-specific diarrhoeal diagnoses were ICD-10 codes A09·9, A09·0, A08·5, A08·4, A05·8, A05·9, A04·9, A04·8, B81·8, K52·8 and K52·9. Death was defined by a discharge status of dead or an entry related to diarrhoea on Thailand's death registry. High cost is referred to admissions costing greater than THB 10,000. Underlying diseases were identified using secondary diagnosis ICD-10 codes for diabetes mellitus (E10–E14, O24·4, N08·3, H36·0, H28·0, G59·0, G63·2, M14·2), hypertension (I10–I15), chronic kidney disease (N18·3–N18·6), chronic obstructive pulmonary disease (J44), stroke (I60–I69) and COVID-19 (U07·1, U10·9). Complications considered were bacterial sepsis (ICD-10 A40, A41), dialysis (ICD-9 39·95) and respiratory failure (ICD-9 96·71, 96·72).

Hospital levels were classified into primary (<150 beds, community hospitals), secondary (151–499 beds, general hospitals) and tertiary hospitals (>500 beds, regional hospitals). Pre-pandemic period refers to admissions between 2014 and 2019, and pandemic period refers to admissions between 2020 and 2022. The Oxford Stringency Index (OSI) is a composite measure of nine policies imposed in response to the COVID-19 pandemic.^12^ Adjusted relative weight (Adj.RW) is a weighted score that reflects the expected resource use for an admission; it was used to calculate reimbursement. Readmission was defined as a subsequent admission for the same patient occurring within 30 days after the prior discharge. Severity was graded as severe in admissions resulting in death or those that were high-cost, had major complications, had LOS ≥5 days or Adj.RW ≥ 0·9; all other admissions were considered moderate.

### Statistical analysis

Categorical variables were presented as frequencies and percentages. Continuous variables were reported as means and standard deviations (SD) or medians and interquartile ranges (IQRs). Age-Standardized Admission Rates (ASRs) of cases per 100,000 population were calculated using the direct method, using the mid-year population from Thailand's Bureau of Registration Administration and the weighting from the World Health Organization’ (WHO) World Standard Population (2000–2025).^13^ Cost in purchasing power parity US dollars ($PPP) was calculated by the cost in THB divided by the PPP conversion factor in the median year.^14^ DALYs were calculated by summing years of life lost (YLL) and years lived with disability (YLD). YLLs were derived from the number of deaths multiplied by the standard life expectancy (WHO standard: 92 years). YLDs were calculated as the product of disease prevalence and disability weights from the Global Burden of Disease (GBD) 2019: 0·188 for moderate and 0·247 for severe diarrhoea.^5^ Trends in specific pathogen diagnoses were visualised using line charts with 3-month moving averages.

To identify risk factors for severe outcomes, we assessed multicollinearity among variables using the variance inflation factor (VIF), then constructed separate multivariable logistic regression models for two binary outcomes, in-hospital death and high-cost admission (defined as > THB 10,000). Predictor variables included in the models were age group, sex, hospital level, region, insurance scheme, specific pathogen type, comorbidities, complications, readmission, and OSI. Results were reported as adjusted odds ratios (aORs) with 95% confidence intervals (CIs) and exact p-values.

To evaluate the impact of rotavirus vaccination among children younger than 5 years of age, we employed three analytic approaches: (i) A two-way fixed-effects difference-in-differences (DID) model,^15^ was used to compare rotavirus admissions against other pathogens before and after vaccine introduction in 2020; (ii) A multivariable negative binomial regression (NBR) model adjusted for sex, hospital level, region, insurance scheme, COVID-19 infection, sepsis, respiratory failure, OSI, repeated admissions, and LOS, with results presented as incidence rate ratios (IRRs) and 95% confidence intervals (CIs). (iii) Interrupted time series (ITS) analysis using NBR to model the pre-2020 trend and seasonality and to generate a counterfactual prediction of post-2020 rotavirus admissions assuming no vaccine introduction; observed admissions were compared with this counterfactual. All analyses were performed using RStudio version 2025·05·0 with R version 4·5·0. A p-value <0·05 was considered statistically significant.

### Ethics statement

This study was approved by the Ethics Committee of the Faculty of Tropical Medicine, Mahidol University (MUTM 2025-055-01). The analysis used anonymised routinely collected administrative claims data provided by the NHSO, Thailand. The authors had access only to de-identified records, and no direct identifiers were available to the authors. Informed consent was waived because the study involved secondary analysis of anonymised data.

### Role of the funding source

The funders had no role in study design, data collection, data analysis, interpretation, or writing of the report.

## Results

After excluding records with missing data (1), inconsistent death dates (26), duplicate entries (565) and readmissions (32,142), a total of 3,041,699 hospitalized admissions were included in the final analysis.

From the pre-pandemic to the pandemic period, mean annual admissions decrease 30·9% (from 376,712 ± 27,133 to 260,475 ± 49,189; ASR declined from 686·5 to 474·5 per 100,000; Notably, admissions for children under five decrease 45%, from 124,665 to 68,532. Patients’ median age was 22 years (IQR 3–61); 1,651,086 (54·3%) were female. The mean number of diarrhoeal episodes was 1·4 ± 1·2, mean LOS was 2·5 ± 2·6 days, which slightly increased from 2·4 days to 2·6 days during pandemic period. Children under five had most admissions (31·4%), while adults above 60 years comprised 25·7% of admissions, accounted for the most deaths (64·6%) and had the longest stays and highest costs (Table 1 and Table 2).

**Table 1:** Characteristics and burden of all diarrheal admissions.

Variable	Admission (%)	LOS ± SD	Cost ± SD ($PPP)^a^	Death (CFR)	DALYs
Overall	3,041,699 (100%)	2·5 ± 2·6	415 ± 1238	18,782 (0·6%)	1,124,618
Sex					
Female	1,651,086 (54·3%)	2·4 ± 2·5	408 ± 1408	9440 (0·6%)	559,961
Male	1,390,613 (45·7%)	2·5 ± 2·7	423 ± 1000	9342 (0·7%)	564,657
Age group					
<5	953,586 (31·4%)	2·5 ± 2·1	326 ± 1112	339 (0·04%)	216,100
5–18	496,548 (16·3%)	2·0 ± 1·6	293 ± 1088	187 (0·04%)	110,351
18–40	304,788 (10%)	2·1 ± 2·4	403 ± 1021	1225 (0·4%)	132,871
40–60	505,365 (16·6%)	2·5 ± 2·8	488 ± 1135	4907 (1·0%)	298,013
>60	781,412 (25·7%)	2·8 ± 3·3	559 ± 1557	12,124 (1·6%)	367,284
Hospital level					
Primary	2,280,758 (75·0%)	2·3 ± 1·9	336 ± 900	5649 (0·2%)	584,216
Secondary	473,793 (15·6%)	2·9 ± 3·4	591 ± 1789	7588 (1·6%)	315,396
Tertiary	256,723 (8·4%)	3·0 ± 4·6	772 ± 2157	5463 (2·1%)	216,589
Region					
Northeastern	1,276,861 (42%)	2·3 ± 2·1	373 ± 1129	5948 (0·5%)	423,985
Central	636,710 (20·9%)	2·7 ± 3·4	537 ± 1852	6355 (1·0%)	295,170
Southern	500,351 (16·4%)	2·4 ± 2·2	346 ± 812	1660 (0·3%)	148,609
Northern	286,060 (9·4%)	2·5 ± 2·6	429 ± 904	2024 (0·7%)	111,416
Eastern	182,040 (6%)	2·6 ± 2·9	458 ± 988	1671 (0·9%)	84,009
Western	159,535 (5·2%)	2·6 ± 2·5	408 ± 783	1124 (0·7%)	61,398
Underlying diseases					
DM	248,524 (8·2%)	2·7 ± 2·8	529 ± 1091	2993 (1·2%)	125,617
HT	102,497 (3·4%)	2·4 ± 2·4	426 ± 779	626 (0·6%)	31,178
COVID	6848 (0·2%)	4·9 ± 3·8	1438 ± 2312	4 (0·1%)	1708
CKD	9414 (0·3%)	2·5 ± 2·0	446 ± 559	93 (1·0%)	4209
COPD	7949 (0·3%)	2·7 ± 2·5	482 ± 1318	64 (0·8%)	2624
CVD	6127 (0·2%)	2·6 ± 2·6	520 ± 1114	125 (2·0%)	3882
Stroke	3413 (0·1%)	2·8 ± 2·7	519 ± 806	67 (2·0%)	1993
CHF	1054 (0·03%)	3·8 ± 4·1	845 ± 2486	30 (2·8%)	1161
Complications					
Sepsis	34,880 (1·1%)	5·6 ± 6·8	1518 ± 3506	2800 (8·0%)	87,731
Dialysis	5581 (0·2%)	9·6 ± 12·2	4730 ± 8512	962 (17·2%)	30,320
Respiratory failure	19,879 (0·7%)	8·8 ± 13·5	5086 ± 7656	10,179 (51·2%)	310,104
Other					
Pandemic period	387,235 (12·7%)	2·7 ± 3·0	616 ± 1464	3777 (1·0%)	179,488
Readmission	61,387 (2%)	3·4 ± 3·9	555 ± 1262	630 (1·0%)	29,366

LOS, length of stay; SD, standard deviation; CF, case fatality rate; DALYs, disability-adjusted life years; DM, diabetes mellitus; HT, hypertension; COVID, coronavirus disease; CKD, chronic kidney disease; COPD, chronic obstructive pulmonary disease; CVD, cardiovascular disease; CHF, congestive heart failure.

a 1 $PPP = 12·15 baht.

**Table 2:** Characteristics and burden of diarrhoeal admissions by COVID-19 pandemic period.

Variable	Pre-pandemic period (2014–2019)					Pandemic period (2020–2022)				
	Mean Admission per year ± SD	LOS ± SD	Cost ± SD ($PPP)^a^	Mean Death per year ± SD	Mean DALYs per year ± SD	Mean Admission per year ± SD	LOS ± SD	Cost ± SD ($PPP)^a^	Mean Death per year ± SD	Mean DALYs per year ± SD
Overall	376,712 ± 27,133	2·4 ± 2·5	372 ± 1209	2047 ± 176	132,366 ± 7831	260,475 ± 49,189	2·6 ± 2·8	539 ± 1311	2167 ± 47	110,140 ± 11,266
Sex										
Female	203,740 ± 14,193	2·4 ± 2·4	368 ± 1452	1038 ± 67	66,478 ± 2964	142,882 ± 27,197	2·5 ± 2·8	523 ± 1269	1070 ± 27	53,697 ± 5935
Male	172,972 ± 12,961	2·5 ± 2·6	378 ± 839	1009 ± 111	65,888 ± 5017	117,594 ± 22,028	2·7 ± 2·9	558 ± 1360	1096 ± 24	56,443 ± 5333
Age group										
<5	124,665 ± 10,616	2·5 ± 2·0	307 ± 1193	43 ± 8	28,108 ± 2208	68,532 ± 16,829	2·5 ± 2·3	397 ± 741	27 ± 13	15,818 ± 3885
5–18	61,176 ± 8825	2·0 ± 1·6	268 ± 1169	22 ± 4	13,512 ± 1892	43,163 ± 8771	2·0 ± 1·8	363 ± 812	18 ± 5	9760 ± 1982
18–40	37,011 ± 1887	2·1 ± 2·4	356 ± 803	142 ± 15	15,692 ± 1009	27,574 ± 4978	2·3 ± 2·5	529 ± 1445	124 ± 12	12,906 ± 1676
40–60	61,236 ± 2742	2·4 ± 2·7	431 ± 944	543 ± 50	34,032 ± 1889	45,984 ± 8560	2·7 ± 3·0	639 ± 1521	550 ± 25	31,274 ± 2630
>60	92,624 ± 5218	2·7 ± 3·2	497 ± 1500	1297 ± 133	41,023 ± 3107	75,222 ± 11,885	3·0 ± 3·6	711 ± 1680	1448 ± 21	40,382 ± 1925
Hospital level										
Primary	280,972 ± 22,760	2·3 ± 1·9	308 ± 974	570 ± 103	69,198 ± 5868	198,310 ± 39,748	2·4 ± 2·1	417 ± 639	744 ± 29	56,342 ± 7061
Secondary	59,889 ± 3553	2·8 ± 3·2	511 ± 1673	840 ± 66	36,546 ± 1882	38,153 ± 6563	3·1 ± 4·0	843 ± 2095	848 ± 48	32,041 ± 2512
Tertiary	32,182 ± 1481	3·0 ± 4·5	657 ± 1835	628 ± 43	25,612 ± 1387	21,209 ± 3378	3·2 ± 4·9	1120 ± 2895	566 ± 6	20,972 ± 1606
Region										
Northeastern	152,430 ± 14,472	2·3 ± 2·0	334 ± 1138	628 ± 76	48,559 ± 4197	120,760 ± 20,107	2·4 ± 2·4	474 ± 1100	727 ± 12	44,210 ± 4220
Central	82,114 ± 5819	2·6 ± 3·2	483 ± 1824	696 ± 63	35,244 ± 2051	48,008 ± 10,813	3·0 ± 3·8	722 ± 1933	726 ± 14	27,903 ± 2620
Southern	63,809 ± 2866	2·3 ± 2·2	309 ± 672	189 ± 23	18,251 ± 958	39,166 ± 9505	2·5 ± 2·3	465 ± 1149	176 ± 12	13,035 ± 2332
Northern	34,953 ± 2465	2·5 ± 2·5	387 ± 849	226 ± 20	13,054 ± 503	25,447 ± 3284	2·6 ± 2·8	544 ± 1033	222 ± 19	11,030 ± 1015
Eastern	23,643 ± 1684	2·6 ± 2·8	398 ± 824	188 ± 9	10,150 ± 500	13,395 ± 2969	2·9 ± 3·2	667 ± 1404	182 ± 2	7703 ± 730
Western	19,746 ± 1613	2·5 ± 2·4	362 ± 676	120 ± 13	7105 ± 638	13,686 ± 2774	2·7 ± 2·8	542 ± 1019	134 ± 8	6255 ± 692
Underlying diseases										
DM	28,597 ± 2491	2·7 ± 2·8	478 ± 923	316 ± 27	13,896 ± 1150	25,647 ± 3721	2·8 ± 2·7	643 ± 1388	367 ± 21	14,080 ± 1291
HT	12,164 ± 1605	2·4 ± 2·2	385 ± 636	68 ± 12	3569 ± 429	9837 ± 2276	2·6 ± 2·7	529 ± 1045	73 ± 8	3255 ± 393
COVID	0 ± 0	NA	NA	0 ± 0	0 ± 0	2283 ± 2937	4·9 ± 3·8	1438 ± 2312	1 ± 2	569 ± 737
CKD	1093 ± 320	2·5 ± 2·1	421 ± 547	10 ± 3	447 ± 118	952 ± 221	2·5 ± 2·0	502 ± 582	12 ± 2	508 ± 94
COPD	1008 ± 48	2·6 ± 2·3	438 ± 551	8 ± 1	335 ± 25	633 ± 166	2·7 ± 3·0	622 ± 2507	5 ± 3	204 ± 80
CVD	741 ± 85	2·6 ± 2·7	483 ± 1088	13 ± 3	442 ± 82	561 ± 122	2·7 ± 2·4	618 ± 1174	16 ± 4	409 ± 35
Stroke	388 ± 61	2·8 ± 2·6	475 ± 634	8 ± 2	227 ± 49	362 ± 49	2·9 ± 2·9	614 ± 1081	7 ± 4	210 ± 112
CHF	128 ± 7	3·8 ± 4·3	823 ± 2775	4 ± 2	164 ± 73	96 ± 1	4·0 ± 3·6	902 ± 1462	1 ± 1	58 ± 13
Complications										
Sepsis	3400 ± 269	5·6 ± 6·8	1399 ± 2951	297 ± 23	9343 ± 620	4827 ± 596	5·7 ± 6·8	1686 ± 4157	340 ± 27	10,558 ± 806
Dialysis	546 ± 97	9·6 ± 11·3	4430 ± 6981	95 ± 8	3007 ± 294	769 ± 118	9·5 ± 13·3	5155 ± 10,289	130 ± 5	4092 ± 416
Respiratory failure	2136 ± 198	8·5 ± 13·7	4593 ± 6850	1144 ± 74	35,076 ± 2407	2354 ± 100	9·3 ± 13·2	5980 ± 8866	1105 ± 54	33,217 ± 1432
Other characteristics										
Readmission	7710 ± 625	3·4 ± 3·7	511 ± 1192	71 ± 7	3454 ± 238	5043 ± 1318	3·6 ± 4·7	690 ± 1447	68 ± 16	2881 ± 527

LOS, length of stay; SD, standard deviation; CF, case fatality rate; DALYs, disability-adjusted life years; DM, diabetes mellitus; HT, hypertension; COVID, coronavirus disease; CKD, chronic kidney disease; COPD, chronic obstructive pulmonary disease; CVD, cardiovascular disease; CHF, congestive heart failure. The mean of Admission, Death, and DALYs by year. The mean of LOS and cost by all admissions.

a 1 $PPP = 12·15 baht.

Moderate and severe cases accounted for 80·7% (2,520,094) and 19·3% (602,243), respectively. The total diarrheal-related death was 18,782 with CFR was 0·6%, increasing with age from 0·04% (<18 years) to 1·6% (>60 years). Mean annual deaths rose from 2047 ± 176 pre-pandemic to 2167 ± 47 during the pandemic, with the CFR increasing from 0·5% to 0·8%. Diabetes and hypertension showed CFRs of 1·2 and 0·6, respectively (Supplementary Figures S5 and S7).

Total admission costs increased from approximately THB 1·5 billion in 2014 to 2·0 billion in 2019, before decreasing to THB 1·8 billion in 2022. The mean cost per admission increased by 45%, rising from $PPP 372 (THB 4524) during pre-pandemic period to $PPP 539 (THB 6546) during the pandemic period. The proportion of high-cost admissions increased steadily from 2014 to 2022, rising from 4·8%, 5·9%, 7·2%, and 9·6% to reach 14·8%, respectively. The mean Adj.RW was 0·4 ± 0·5 (Supplementary Figures S8 and S9).

Total DALYs were 1,165,844, comprising 543,312 YLLs and 622,531 YLDs. The mean annual DALYs between the pre-pandemic and pandemic periods decreased by 17% (from 132,366 to 110,140). Additionally, mean annual DALYs for children under five decreased by 44%, falling from 28,108 to 15,818. Both costs and mortality increased with the level of care, rising from primary to tertiary hospitals. Regionally, the Northeast recorded the highest number of admissions, while the Central region had the highest mean costs and the greatest number of deaths (Table 1, Table 2, and Supplementary Figure S10).

Non-specific diarrhoeal diagnoses were associated with 2,970,033 admissions (97·6%). Among specific diagnoses, *Salmonella* infections and *C. difficile* enterocolitis were responsible for the longest mean LOS 7·6 and 10·5 days, respectively, and the highest mean costs per admission $PPP 1881 and 3586 respectively. *Salmonella* infections also had highest death (3·5%) and the highest DALYs (25,117) (Table 3 and Supplementary Figure S6).

**Table 3:** Characteristics and burden of non-specific and specific diagnoses of all admissions.

ICD-10 code	Diagnosis	Admission (%)	LOS ± SD	Cost ± SD ($PPP)^a^	Death (%)	DALYs
Non-specific diagnosis						
A099	Gastroenteritis and colitis of unspecified origin	1,864,165 (61·3%)	2·3 ± 2·2	378 ± 1031	8095 (0·4%)	588,935
A090	Other and unspecified gastroenteritis and colitis of infectious origin	685,112 (22·5%)	2·8 ± 2·8	488 ± 1130	8518 (1·2%)	371,927
A059	Bacterial foodborne intoxication, unspecified	120,046 (3·9%)	2·2 ± 1·6	306 ± 1933	28 (0·02%)	24,337
A084	Viral intestinal infection, unspecified	119,265 (3·9%)	1·5 ± 1·5	235 ± 364	62 (0·1%)	24,604
A049	Bacterial intestinal infection, unspecified	105,284 (3·5%)	2·8 ± 2·2	414 ± 2378	437 (0·4%)	32,169
K529	Noninfective gastroenteritis and colitis, unspecified	53,132 (1·7%)	3·0 ± 4·6	667 ± 1523	482 (0·9%)	26,226
Specific diagnosis						
A010-A014	Typhoid and paratyphoid	19,101 (0·6%)	3·8 ± 2·5	512 ± 1076	29 (0·2%)	4921
A020-A022, A029	*Salmonella*	15,535 (0·5%)	7·5 ± 8·6	1881 ± 4569	666 (4·3%)	25,117
A060-A063	Amoeba	11,625 (0·4%)	3·1 ± 2·6	451 ± 701	45 (0·4%)	3498
A080	Rotavirus	6498 (0·2%)	3·1 ± 2·2	654 ± 998	5 (0·08%)	1793
B780, B789	*Strongyloides*	5607 (0·2%)	4·1 ± 3·8	804 ± 1439	103 (1·8%)	4035
A040-A044, B962	*E. coli*	3121 (0·1%)	4·4 ± 3·5	702 ± 1087	24 (0·8%)	1318
K5220	Cow milk allergy	2877 (0·1%)	3·7 ± 10·1	660 ± 2453	5 (0·2%)	840
A047	*Clostridioides difficile* enterocolitis	1519 (0%)	10·5 ± 12·8	3586 ± 5981	122 (8·0%)	3500
B770	Ascariasis	1003 (0%)	2·9 ± 2·1	392 ± 666	1 (0·10%)	282
A053	*V. parahaemolyticus*	713 (0%)	2·8 ± 3·4	604 ± 1460	12 (1·7%)	507

LOS, length of stay; SD, standard deviation; DALYs, disability-adjusted life years; ICD-10, International Classification of Disease Codes, 10th Edition.

a 1 $PPP = 12·15 baht.

**Fig. 1:**
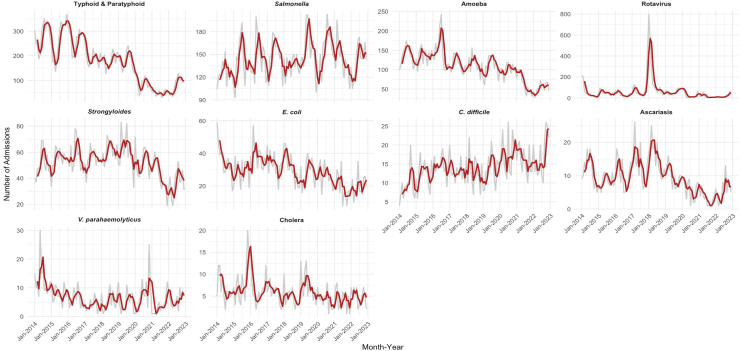
**Monthly diarrhoeal admission trends by most diagnosed pathogens in Thailand, 2014**–**2023.** Grey lines show the observed monthly number of hospital admissions, and red lines show 3-month moving averages to highlight trends. Each panel represents a different pathogen, and y-axis scales differ across panels.

Monthly trends in specific diarrhoeal pathogens showed distinct seasonal and temporal variations Typhoid and paratyphoid, Amoeba, *E. coli*, ascariasis, showed declining trends. *Salmonella* and *Strongyloides* displayed periodic fluctuations. Rotavirus showed a sharp peak in early 2018 *C. difficile* admissions increased steadily over time, while *Vibrio parahaemolyticus* and cholera showed variable but relatively stable trends (Fig. 1).

Significant risk factors for increased mortality in both the pre-pandemic and pandemic periods were older age, male sex, readmission and admission to secondary, tertiary, or central region hospitals. Comorbidities including Diabetes, Hypertension, and COVID-19 showed lower odds while pathogens, *C. difficile* and *Salmonella* showed increased odds of mortality. All complications (sepsis, respiratory failure, dialysis) significantly increased the odds of mortality. Risk factors for high-cost admission were similar to those for mortality. Whild CSMBS scheme and increase LOS were associated with higher odds of high-cost admission, they were associated with lower odds of mortality (Table 4 and Supplementary Table S1).

**Table 4:** Adjusted odds ratio (aOR) of risk factors for high-cost and death among diarrheal admissions by pre-pandemic and pandemic period.

Variable	Pre-pandemic period (2014–1019)				Pandemic period (2020–2022)			
	aOR (95% CI) for high-cost admission	p-value	aOR (95% CI) for diarrhoeal-associated death	p-value	aOR (95% CI) for high-cost admission	p-value	aOR (95% CI) for diarrhoeal-associated death	p-value
Age group								
<5	Reference		Reference		Reference		Reference	
5–18	1·91 (1·86–1·97)^a^	<0·0001	1·25 (1·02–1·55)^a^	0·035	2·28 (2·19–2·36)^a^	<0·0001	1·19 (0·84–1·69)	0·33
18–40	4·61 (4·48–4·73)^a^	<0·0001	9·97 (8·62–11·52)^a^	<0·0001	5·17 (4·98–5·37)^a^	<0·0001	10·07 (7·86–12·89)^a^	<0·0001
40–60	4·81 (4·70–4·93)^a^	<0·0001	17·59 (15·41–20·07)^a^	<0·0001	5·32 (5·15–5·49)^a^	<0·0001	20·36 (16·17–25·63)^a^	<0·0001
>60	4·97 (4·86–5·08)^a^	<0·0001	28·32 (24·89–32·23)^a^	<0·0001	5·48 (5·32–5·65)^a^	<0·0001	35·23 (28·09–44·19)^a^	<0·0001
Sex								
Female	Reference		Reference		Reference		Reference	
Male	1·10 (1·09–1·12)^a^	<0·0001	1·40 (1·34–1·46)^a^	<0·0001	1·09 (1·07–1·11)^a^	<0·0001	1·37 (1·30–1·46)^a^	<0·0001
Hospital level								
Primary	Reference		Reference		Reference		Reference	
Secondary	2·79 (2·74–2·84)^a^	<0·0001	2·89 (2·73–3·05)^a^	<0·0001	4·51 (4·40–4·61)^a^	<0·0001	2·25 (2·09–2·41)^a^	<0·0001
Tertiary	4·07 (3·98–4·15)^a^	<0·0001	2·84 (2·66–3·02)^a^	<0·0001	7·79 (7·57–8·01)^a^	<0·0001	2·26 (2·08–2·46)^a^	<0·0001
Region								
Southern	Reference		Reference		Reference		Reference	
Northeastern	1·48 (1·44–1·52)^a^	<0·0001	0·89 (0·82–0·97)^a^	0·006	1·21 (1·18–1·25)^a^	<0·0001	0·89 (0·79–0·99)^a^	0·029
Central	3·49 (3·41–3·58)^a^	<0·0001	2·06 (1·90–2·23)^a^	<0·0001	1·60 (1·55–1·65)^a^	<0·0001	2·35 (2·11–2·63)^a^	<0·0001
Northern	2·12 (2·06–2·19)^a^	<0·0001	1·44 (1·31–1·58)^a^	<0·0001	1·49 (1·43–1·55)^a^	<0·0001	1·34 (1·18–1·53)^a^	<0·0001
Eastern	1·57 (1·52–1·63)^a^	<0·0001	1·95 (1·76–2·16)^a^	<0·0001	1·75 (1·68–1·83)^a^	<0·0001	2·13 (1·85–2·46)^a^	<0·0001
Western	1·24 (1·19–1·29)^a^	<0·0001	1·45 (1·30–1·63)^a^	<0·0001	0·93 (0·88–0·97)^a^	0·002	1·63 (1·40–1·90)^a^	<0·0001
Insurance								
UCS	Reference		Reference		Reference		Reference	
CSMBS	1·26 (1·22–1·29)^a^	<0·0001	0·76 (0·68–0·84)^a^	<0·0001	1·04 (1·00–1·08)^a^	0·036	0·74 (0·65–0·85)^a^	<0·0001
Specific Pathogens								
Typhoid/Paratyphoid	0·57 (0·53–0·62)^a^	<0·0001	0·47 (0·29–0·74)^a^	0·001	0·85 (0·75–0·96)^a^	0·010	0·52 (0·25–1·08)	0·08
*Salmonella*	1·26 (1·18–1·35)^a^	<0·0001	2·15 (1·85–2·50)^a^	<0·0001	1·22 (1·12–1·34)^a^	<0·0001	1·97 (1·65–2·36)^a^	<0·0001
Rotavirus	3·51 (3·25–3·78)^a^	<0·0001	0·43 (0·14–1·35)	0·15	1·38 (1·11–1·73)^a^	0·004	2·08 (0·50–8·55)	0·31
*E. coli*	1·13 (0·96–1·32)	0·14	1·32 (0·70–2·49)	0·39	1·18 (0·95–1·47)	0·14	0·60 (0·24–1·53)	0·28
*C. difficile*	3·26 (2·60–4·09)^a^	<0·0001	2·08 (1·44–3·00)^a^	<0·0001	2·66 (1·96–3·60)^a^	<0·0001	2·80 (1·93–4·06)^a^	<0·0001
Cholera	1·78 (1·28–2·47)^a^	0·0005	0·43 (0·16–1·10)	0·08	0·78 (0·43–1·42)	0·42	1·20 (0·38–3·76)	0·76
*Shigella*	1·71 (1·25–2·34)^a^	0·0009	0·61 (0·20–1·87)	0·39	1·67 (0·90–3·11)	0·10	0·71 (0·17–3·01)	0·64
Norovirus	3·15 (1·78–5·56)^a^	<0·0001	–		1·89 (0·94–3·82)	0·08	1·57 (0·18–13·94)	0·68
Comorbidities								
DM	0·95 (0·92–0·97)^a^	<0·0001	0·92 (0·86–0·98)^a^	0·006	0·89 (0·87–0·92)^a^	<0·0001	0·89 (0·82–0·97)^a^	0·005
HT	0·75 (0·72–0·78)^a^	<0·0001	0·66 (0·59–0·74)^a^	<0·0001	0·74 (0·71–0·78)^a^	<0·0001	0·60 (0·52–0·70)^a^	<0·0001
CKD	0·85 (0·76–0·95)^a^	0·004	1·24 (0·92–1·66)	0·15	0·65 (0·56–0·76)^a^	<0·0001	1·23 (0·85–1·77)	0·27
COPD	0·88 (0·78–0·99)^a^	0·036	0·77 (0·56–1·07)	0·12	1·01 (0·85–1·19)	0·94	0·56 (0·32–0·98)^a^	0·044
Stroke	0·88 (0·74–1·06)	0·17	1·88 (1·30–2·71)^a^	0·0008	0·93 (0·75–1·15)	0·49	1·49 (0·91–2·46)	0·11
COVID-19	–		–	–	4·42 (4·10–4·77)^a^	<0·0001	0·29 (0·11–0·77)^a^	0·014
Complications								
Sepsis	1·94 (1·85–2·03)^a^	<0·0001	3·55 (3·26–3·86)^a^	<0·0001	1·73 (1·65–1·82)^a^	<0·0001	2·46 (2·24–2·71)^a^	<0·0001
Dialysis	6·27 (5·54–7·09)^a^	<0·0001	1·07 (0·94–1·23)	0·31	4·39 (3·77–5·10)^a^	<0·0001	1·36 (1·17–1·60)^a^	0·0001
Respiratory failure	83·20 (78·13–88·6)^a^	<0·0001	135·5 (128–143)^a^	<0·0001	74·34 (66·2–83·5)^a^	<0·0001	74·72 (69·3–80·6)^a^	<0·0001
Other factors								
Readmission	0·88 (0·85–0·92)^a^	<0·0001	2·31 (2·06–2·60)^a^	<0·0001	0·85 (0·80–0·91)^a^	<0·0001	2·14 (1·83–2·51)^a^	<0·0001
OSI (per unit)	–		–	–	1·003 (1·002–1·004)^a^	<0·0001	1·001 (0·99–1·00)	0·18
Length of stay	2·29 (2·28–2·30)^a^	<0·0001	0·98 (0·97–0·98)^a^	<0·0001	2·45 (2·43–2·46)^a^	<0·0001	0·98 (0·97–0·98)^a^	<0·0001

UCS, Universal Coverage Scheme; CSMBS, Civil Servant Medical Benefits Scheme; DM, diabetes mellitus; HT, hypertension; COVID, coronavirus disease; CKD, chronic kidney disease; COPD, chronic obstructive pulmonary disease; CVD, cardiovascular disease; CHF, congestive heart failure; OSI, Oxford Stringency Index.

a p-value <0·05.

Children younger than 5 years of age accounted for the greatest proportion of admissions (31·4%). Among specific diagnoses in children, Salmonella infections were associated with the most severe outcomes, longest mean LOS), highest mean cost, and greatest number of deaths. Rotavirus accounted 1·5% of deaths among specific pathogen groups, and a total burden of 1531 DALYs. Risk factors among children for high-cost admission and mortality are summarised in Supplementary Tables S1–S4.

**Fig. 2:**
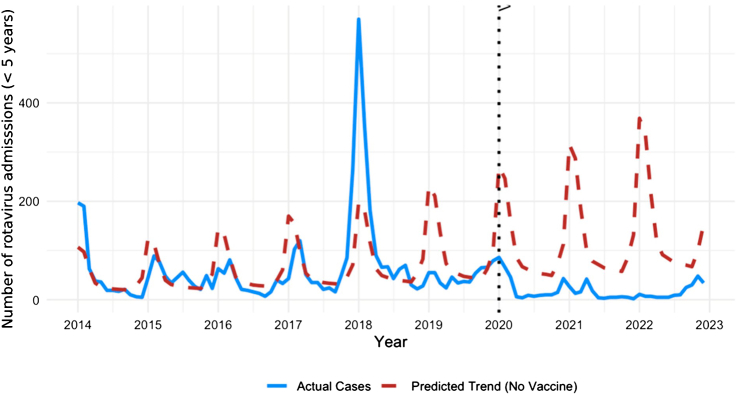
**Interrupted time-series analysis of monthly rotavirus admissions among children aged under 5 years in Thailand, 2014**–**2023.** Observed monthly admissions are shown by the blue solid line, whereas the red dashed line represents the model-based counterfactual trend assuming no vaccine introduction. The vertical dotted line indicates the 2020 introduction of rotavirus vaccine into Thailand's National Immunization Program.

The DID model estimated that the rotavirus vaccine was associated with a reduction of 38·2 (standard error = 2·261, p < 0·001) admissions per month for rotavirus compared with all other pathogens. The NBR result showed that for every 1% increase in monthly vaccine coverage, the incidence rate of rotavirus-related admissions decreased by 2·0% (IRR = 0·980, 95% CI: 0·974–0·986, p < 0·001). The ITS analysis with NBR showed a pronounced seasonal peak for rotavirus in early 2018. Following the introduction of rotavirus vaccine into Thailand's National Immunization Program in 2020, observed admissions declined and remained consistently lower than the predicted counterfactual trend assuming no rotavirus vaccination (Fig. 2).

## Discussion

Our analysis of over 3 million hospitalisations revealed a U-shaped epidemiological burden of acute diarrhoea, primarily affecting children younger than 5 years of age in admission volume and adults older than 60 years of age in mortality and costs. Addressing our objectives, we found that while the COVID-19 pandemic significantly reduced total diarrhoeal admissions, it paradoxically increased per-case severity, overall hospital expenditures, and the case fatality ratio. Pathogen trends indicated a steady rise in *C. difficile*. Furthermore, our multivariable models identified systemic clinical complications (respiratory failure, dialysis, and sepsis), older age, hospital readmission, and severe bacterial infections (*Salmonella* and *C. difficile*) as the strongest predictors of mortality and high-cost admissions. Finally, our subgroup analysis demonstrated a significant reduction in rotavirus admissions among children younger than 5 years of age following rotavirus vaccine introduction.

The annual admissions for diarrhoeal disease decreased by nearly one-third during the COVID-19 pandemic, a finding that is consistent with findings from China and Ghana.^16^^,^^17^ It may be attributable to nonpharmaceutical interventions, which aimed to reduce admissions and avoid overwhelming the hospital capacity, COVID-19 restrictions, as measured by the OSI, were associated with increase in higher-cost and fatal diarrhoeal admissions, probably due to delayed care-seeking, hospitals being under strain from resource diversion, and a shift towards a more severity threshold for admission.^18^

Thailand's ASR of diarrhoeal admissions in 2014–2018 (686·5 per 100,000) was lower than the GBD 2021 estimate of the age-standardised prevalence rate for Southeast Asia 820·5 per 100,000.^19^ This gap is expected because our estimate reflects only hospital admissions (the severe end of the spectrum and influenced by admission thresholds and access), whereas the GBD prevalence metric captures all diarrhoeal episodes in the population.

The mean LOS was 2·4 days. This pattern is consistent with findings from a 2010 study in Thailand,^7^ and is closely comparable to a Vietnam study, where the average length of stay for diarrhoeal admissions is 2·8 days.^20^

The CFR of 0·6% we observed was consistent with the 0·5% observed in Thailand's 2010 hospital data.^7^ This study's diarrhoeal deaths and DALYs were substantially lower than the GBD 2021 model estimates of Thailand, with deaths being 67% and 69% lower and DALYs 41% and 52% lower during the pre-pandemic and pandemic periods, respectively.^21^ This may be because we did not include outpatients or because the GBD model overestimated deaths. We found that children younger than five (31·4%) and adults older than 60 (25·7%) year of age had the highest morbidity, with older people accounting for 64·6% of deaths, aligning with incidence-rate predictions from the XGBoost and SHAP models.^22^ This U-shaped pattern probably reflects underdeveloped immunity in children and immunosenescence in older people.^23^

Our estimated pre-pandemic mean admission cost of 415 $PPP was 20% higher than from the Thailand's 2010 study, which reported 346 $PPP per diarrhoeal admission.^7^ It is also higher when compared with a 2013 Vietnam study which reported US$106·9 per hospitalisation for foodborne diarrhoea and found that indirect costs comprised 51·3% of total costs.^20^ However, cross-country comparisons should be interpreted cautiously because of differences in price year and costing methods (nominal US$ vs PPP). Overall, these findings underscore that diarrhoeal hospitalisations impose a meaningful economic burden in Low and middle-income settings.^19^ The proportion of high-cost admissions increased steadily, from 4·8% in 2014 to 14·8% in 2022. This upward trend probably reflects multiple factors including increasing healthcare prices over time, increased numbers of complex, expensive-to-treat cases during the COVID-19 pandemic, and the known high-cost user phenomenon, where a small proportion of admissions account for a disproportionately large share of healthcare spending. A recent study from Thailand confirmed that 5% of admission accounted for half of all inpatient expenditures.^24^

The odds of both high-cost admission and death increased with age. This was consistent with Thailand-specific and global evidence that older adults experience the greatest mortality and resource use due to diarrhoeal diseases.^5^^,^^7^ Being of male sex was linked with worse hospital outcomes, also in line with prior inpatient analyses in Thailand.^7^ Elevated odds of worse outcomes at higher-level hospitals probably reflect the referral of admissions with complications. The highest odds, seen in the Central region, may be linked to the high concentration of large referral and teaching hospitals in and around Bangkok. Compared with the UCS, CSMBS insurance coverage showed higher odds of high-cost admission but lower odds of death, which is plausible given the more generous benefits and broader access pathways.

Among specific pathogens, *Salmonella* accounted for the most severe outcomes, including the long LOS (7·5 days), highest mean costs, highest mortality (3·3%) and greatest DALY burden with significant odds of high-cost admission (1·24) and death (2·09). This mirrors international data reporting prolonged LOS (6·4–8·4 days) and elevated risk of mortality with *Salmonella*, particularly with invasive or antimicrobial-resistant infections.^25^ (Supplementary Figure S11)

Rotavirus peaked sharply in 2018, in line with national surveillance data documenting a surge in infections caused by serotype G9P [8], which replaced the previously predominant G1P [8] lineage, suggesting a population-level immunity gap. Thailand introduced the rotavirus vaccine into its National Immunization Program in 2020; therefore, future peaks are anticipated to be attenuated, with a shift in the genotype distribution.^26^^,^^27^

*C. difficile* infection (CDI) showed a steady increase over time, consistent with regional and global reports of rising CDI burden in the context of ageing populations, antibiotic exposure, and improved diagnostics.^28^^,^^29^ A Thailand-focused review noted that earlier practice often relied on toxin enzyme immunoassays, which may underestimate CDI, whereas later adoption of more sensitive PCR-based assays could increase case ascertainment over time. Because our data do not capture laboratory ordering or test type, we cannot disentangle true changes in CDI incidence from changes in testing or coding, and we interpret these temporal trends cautiously.^30^ In contrast, typhoid and paratyphoid showed a decreasing trend, consistent with national surveillance data from 2003 to 2014,^31^ and reflecting improvements in water, sanitation, and food safety.^32^ Similar declines were observed for amoebiasis, *E. coli* and ascariasis.

Readmissions were uncommon but clinically important. In multivariable models, readmission status was associated with lower odds of high-cost admission (aOR 0·87, 95% CI 0·84–0·90) but more than double the odds of death (aOR 2·26, 2·06–2·48). This apparent cost-mortality paradox may reflect per-admission costing and clinical pathways. Some readmitted patients may deteriorate and die early before accruing prolonged ICU/ward costs; repeat admissions may involve fewer diagnostic investigations with more targeted treatment; and a proportion of readmissions may be brief returns for residual dehydration or electrolyte disturbances. Nevertheless, readmissions can still capture unresolved infection, reinfection, chronic comorbidity, or frailty, identifying a subgroup at substantially higher mortality risk.

The DID model estimated a significant monthly reduction of 38·2 rotavirus admissions following the introduction of the rotavirus vaccine, while the NBR showed a clear effect of each 1% increase in coverage was associated with a 2·0% decline in admission rates. These findings are consistent with surveillance data in Thailand that showed considerable post-vaccine declines in admissions and attenuated seasonality, particularly among infants,^4^ and with MAL-ED community surveillance identifying rotavirus as a leading attributable cause of diarrhoea in infancy, with lower attributable burden at sites where rotavirus vaccination was used.^33^

This study has limitations. We used retrospective routinely collected administrative claims data, which are subject to diagnostic misclassification and potential changes in coding or testing practices over time. Our dataset included admissions reimbursed under the Universal Coverage Scheme and the Civil Servant Medical Benefit Scheme, covering roughly 80% of Thailand's population, but excluded the Social Security Scheme, which insures much of the private-sector workforce (about 19%). As a result, our estimates likely under-represent diarrhoeal admissions among working-age adults. Nonetheless, because the highest morbidity and mortality burdens occurred in children younger than five years and adults older than 60 years of age, groups predominantly covered by the included schemes, the overall impact on our main conclusions is likely limited. Ethnicity data is not collected in NHSO claims, and the database captures only individuals belonging to Thai nationality; therefore, we could not report or analyse ethnicity-specific patterns. Finally, more than 95% of admissions were coded with non-specific diagnoses, likely reflecting limited aetiological testing in routine care. In Thailand, stool culture remains the predominant diagnostic test and has low yield in unselected diarrhoea (eg, 1·47% positivity in a large Korean cohort),^34^ while US practice guidance summarises typical yields of 1·5%–5·6%.^35^ Stool multiplex PCR is generally available only in larger tertiary or academic centres. Consequently, trends observed for specific pathogens (eg, *Salmonella* and rotavirus) likely represent a clinically selected subset of tested patients, with potentially severe presentations, and should be interpreted cautiously.

This study's strength is that it provides a contemporary, policy-relevant national picture of diarrhoeal admissions in Thailand. By combining ASR, purchasing-power-parity costs, and DALYs calculated using standard WHO/GBD approaches, the study quantifies burden in metrics that are comparable across time and settings. The analytic design further strengthens inference by incorporating time-stratified comparisons (pre-pandemic vs pandemic), multivariable models for death and high-cost admissions, and vaccine impact evaluations for rotavirus.

In conclusion, this study provides evidence to inform priorities for diarrhoeal disease prevention and service planning in Thailand. Continued investment in vaccination, water, sanitation, and hygiene, together with stronger preparedness for severe presentations in high-risk groups, will be important for reducing future burden. Further research should strengthen pathogen-specific surveillance, expand access to molecular diagnostics, and monitor long-term post-pandemic and post-vaccine trends to support more targeted and efficient policy responses.

## Contributors

Conceptualisation: SS, SC, NS, KP and WP. Data curation: SS, KP and WP. Formal analysis: SS, NS and WP. Funding acquisition: WP. Methodology: SS, SC, NS, KP and WP. Supervision: SC, NS, KP and WP. Validation: SS, KP and WP. Data interpretation: SS, SC, NS, KP and WP. Writing—original draft: SS. Writing—review & editing: All authors.

## Data sharing statement

The raw data that support the findings of this study were shared as per the signed contract and regulations by the collaborative initiative between the National Health Security Office (NHSO) and the Gastroenterological Association of Thailand (GAT), and so are not publicly available. However, the aggregate data are made available from the authors and can be accessed through https://github.com/MuMiMaNz/data_diarrhea_thailand_research via registration with email only.

## Declaration of interests

The authors declare no competing interests.
