# Supplementary material for: Burden of diarrhoeal diseases among hospitalised patients in Thailand: a retrospective national database analysis (2014–2022)
Source: Lancet Reg Health Southeast Asia. 2026 Apr 6;48:100761. doi: 10.1016/j.lansea.2026.100761 (PMC13090140; doi:10.1016/j.lansea.2026.100761)
Supplement: Supplementary Figures and Tables [file mmc1.docx]

**Supplementary**

**Table S1 Adjusted (aOR) odds ratio of risk factors among admissions with diarrhoeal illness**

| Variable | aOR (95% CI) for high-cost admission | p-value | aOR (95% CI) for diarrhoeal-associated death | p-value |
| --- | --- | --- | --- | --- |
| **Age group** |  |  |  |  |
| - < 5 | Reference | - | Reference |  |
| - 5–18 | 2·07 (2·03–2·12) * | <0·0001 | 1·22 (1·02–1·46) * | 0·032 |
| - 18–40 | 4·72 (4·62–4·83) * | <0·0001 | 9·86 (8·70–11·17) * | <0·0001 |
| - 40–60 | 4·92 (4·83–5·02) * | <0·0001 | 18·19 (16·23–20·39) * | <0·0001 |
| - > 60 | 5·10 (5·01–5·19) * | <0·0001 | 30·22 (27·02–33·79) * | <0·0001 |
| **Sex** |  |  |  |  |
| - Female | Reference | - | Reference |  |
| - Male | 1·10 (1·09–1·11) * | <0·0001 | 1·39 (1·34–1·44) * | <0·0001 |
| **Hospital level** |  | <0·0001 |  |  |
| - Primary | Reference | - | Reference |  |
| - Secondary | 3·28 (3·23–3·32) * | <0·0001 | 2·64 (2·53–2·76) * | <0·0001 |
| - Tertiary | 5·04 (4·96–5·12) * | <0·0001 | 2·65 (2·52–2·79) * | <0·0001 |
| **Region** |  |  |  |  |
| - Southern | Reference | - | Reference |  |
| - Northeastern | 1·37 (1·34–1·40) * | <0·0001 | 0·89 (0·83–0·95) * | 0·0003 |
| - Central | 2·62 (2·57–2·67) * | <0·0001 | 2·16 (2·02–2·30) * | <0·0001 |
| - Northern | 1·81 (1·77–1·86) * | <0·0001 | 1·41 (1·30–1·52) * | <0·0001 |
| - Eastern | 1·60 (1·56–1·65) * | <0·0001 | 2·00 (1·84–2·17) * | <0·0001 |
| - Western | 1·10 (1·07–1·13) * | <0·0001 | 1·52 (1·38–1·66) * | <0·0001 |
| **Insurance scheme** |  |  |  |  |
| - UCS | Reference | - | Reference |  |
| - CSMBS | 1·17 (1·15–1·20) * | <0·0001 | 0·75 (0·69–0·82) * | <0·0001 |
| **Pathogen** |  |  |  |  |
| - Non-specific | Reference | - | Reference |  |
| - Typhoid and paratyphoid | 0·59 (0·56–0·63) * | <0·0001 | 0·47 (0·32–0·69) * | 0·0001 |
| - *Salmonella* | 1·24 (1·18–1·30) * | <0·0001 | 2·09 (1·86–2·34) * | <0·0001 |
| - Amoeba | 0·71 (0·65–0·78) * | <0·0001 | 0·76 (0·54–1·07) | 0·12 |
| - Rotavirus | 3·16 (2·95–3·40) * | <0·0001 | 0·64 (0·26–1·54) | 0·32 |
| - *Strongyloides* | 0·98 (0·89–1·07) | 0·67 | 1·06 (0·83–1·36) | 0·64 |
| - *E. coli* | 1·14 (1·01–1·30) * | 0·038 | 0·97 (0·57–1·66) | 0·91 |
| - Cow milk allergy | 1·19 (1·01–1·40) * | 0·040 | 0·74 (0·27–2·02) | 0·56 |
| - *C. difficile* | 3·03 (2·53–3·63) * | <0·0001 | 2·48 (1·90–3·23) * | <0·0001 |
| - Ascariasis | 1·34 (0·96–1·87) | 0·09 | 1·11 (0·16–7·95) | 0·92 |
| **Comorbidities** |  |  |  |  |
| - DM | 0·93 (0·92–0·95) * | <0·0001 | 0·91 (0·87–0·96) * | 0·0003 |
| - HT | 0·75 (0·73–0·77) * | <0·0001 | 0·63 (0·58–0·69) * | <0·0001 |
| - CKD | 0·76 (0·69–0·83) * | <0·0001 | 1·25 (0·99–1·57) | 0·06 |
| - COPD | 0·91 (0·83–1·01) | 0·06 | 0·71 (0·53–0·93) * | 0·014 |
| - Stroke | 0·91 (0·79–1·04) | 0·18 | 1·74 (1·30–2·34) * | 0·0002 |
| - COVID-19 | 5·74 (5·33–6·18) * | <0·0001 | 0·34 (0·12–0·90) * | 0·030 |
| **Complications** |  |  |  |  |
| - Sepsis | 1·88 (1·82–1·94) * | <0·0001 | 3·06 (2·88–3·27) * | <0·0001 |
| - Dialysis | 5·60 (5·09–6·15) * | <0·0001 | 1·17 (1·06–1·30) * | 0·003 |
| - Respiratory failure | 74·90 (70·90–79·12) * | <0·0001 | 109·82 (104·98–114·90) * | <0·0001 |
| **Other factors** |  |  |  |  |
| - Readmissions | 0·87 (0·84–0·90) * | <0·0001 | 2·26 (2·06–2·48) * | <0·0001 |
| - OSI (per unit) | 1·02 (1·02–1·02) * | <0·0001 | 1·003 (1·002–1·004) * | <0·0001 |
| - Length of stay (days) | 2·32 (2·31–2·32) * | <0·0001 | 0·98 (0·98–0·98) * | <0·0001 |

* p-value < 0·05, UCS, Universal Coverage Scheme; CSMBS, Civil Servant Medical Benefits Scheme; DM, diabetes mellitus; HT, hypertension; COVID, coronavirus disease; CKD, chronic kidney disease; COPD, chronic obstructive pulmonary disease; OSI, Oxford Stringency Index.

**Table S2 Characteristics and burden of all children under five years old diarrheal admissions**

| Variable | Admission (%) | LOS ± SD | Cost ± SD ($PPP)* | Death (CFR) | DALYs |
| --- | --- | --- | --- | --- | --- |
| **Sex** |  |  |  |  |  |
| - Female | 409,306 (42·9%) | 2·5 ± 2·1 | 325 ± 1,557 | 154 (0.04%) | 93,465 |
| - Male | 544,280 (57·1%) | 2·5 ± 2·1 | 327 ± 587 | 185 (0.03%) | 122,635 |
| **Hospital Level** |  |  |  |  |  |
| - Primary | 657,667 (69%) | 2·4 ± 1·5 | 289 ± 1,182 | 72 (0.01%) | 133,548 |
| - Secondary | 180,429 (18·9%) | 2·7 ± 2·1 | 368 ± 497 | 132 (0.07%) | 47,397 |
| - Tertiary | 105,202 (11%) | 2·9 ± 4·1 | 471 ± 1,363 | 135 (0.1%) | 33,107 |
| **Region** |  |  |  |  |  |
| - Northeastern | 356,099 (37·3%) | 2·4 ± 1·7 | 293 ± 1,596 | 81 (0.02%) | 76,111 |
| - Central | 216,592 (22·7%) | 2·6 ± 2·7 | 398 ± 887 | 95 (0.04%) | 51,186 |
| - Southern | 176,919 (18·6%) | 2·5 ± 2·0 | 302 ± 560 | 81 (0.05%) | 41,669 |
| - Northern | 90,157 (9·5%) | 2·5 ± 2·2 | 321 ± 476 | 20 (0.02%) | 19,345 |
| - Eastern | 60,590 (6·4%) | 2·6 ± 2·1 | 338 ± 501 | 32 (0.05%) | 14,693 |
| - Western | 53,228 (5·6%) | 2·6 ± 1·7 | 333 ± 405 | 30 (0.06%) | 13,095 |
| **Complications** |  |  |  |  |  |
| - Sepsis | 2,269 (0·2%) | 6·7 ± 8·8 | 1,260 ± 2,574 | 57 (2.5%) | 5,775 |
| - Dialysis | 8 (0%) | 16·8 ± 17·4 | 12,862 ± 17,637 | 4 (50.0%) | 362 |
| - Respiratory failure | 839 (0·1%) | 14·6 ± 22·0 | 6,628 ± 10,897 | 218 (26.0%) | 20,152 |
| **Other characteristics** |  |  |  |  |  |
| - Pandemic period | 98,411 (10·3%) | 2·6 ± 2·4 | 447 ± 899 | 51 (0.05%) | 23,942 |
| - Readmission | 23,371 (2·5%) | 3·2 ± 3·8 | 401 ± 757 | 11 (0.05%) | 5,670 |

*1 $PPP = 12·15 baht; LOS, length of stay; SD, standard deviation; CFR, case fatality rate; DALYs, disability-adjusted life years.

**Table S3 Characteristics and burden of non-specific and specific diagnoses of
all children under five years old admissions**

| ICD-10 code | Diagnosis | Admission (%) | LOS ± SD | Cost ± SD ($PPP)* | Death (%) | DALYs |
| --- | --- | --- | --- | --- | --- | --- |
| **Non-specific diagnosis** | | | | | | |
| - A099 | Gastroenteritis and colitis of unspecified origin | 614,931 (64·5%) | 2·4 ± 1·7 | 310 ± 1,263 | 185 (54·6%) | 135,827 |
| - A090 | Other and unspecified gastroenteritis and colitis of infectious origin | 167,460 (17·6%) | 2·8 ± 2·0 | 352 ± 481 | 96 (28·3%) | 41,559 |
| - A084 | Viral intestinal infection, unspecified | 81,915 (8·6%) | 2·2 ± 1·4 | 288 ± 353 | 7 (2·1%) | 16,381 |
| - A049 | Bacterial intestinal infection, unspecified | 31,368 (3·3%) | 2·9 ± 1·6 | 348 ± 306 | 6 (1·8%) | 6,697 |
| - A059 | Bacterial foodborne intoxication, unspecified | 17,765 (1·9%) | 1·7 ± 1·6 | 224 ± 197 | 0 (0%) | 3,376 |
| **Specific diagnosis** | | | | | | |
| - A020- A022, A029 | *Salmonella* | 5,879 (0·6%) | 6·7 ± 8·0 | 1,177 ± 3,148 | 18 (5·3%) | 2,956 |
| - A080 | Rotavirus | 5,233 (0·5%) | 3·2 ± 2·2 | 644 ± 1,015 | 5 (1·5%) | 1,531 |
| - A060-A063 | Amoeba | 3,436 (0·4%) | 3·1 ± 1·8 | 371 ± 257 | 1 (0·3%) | 770 |
| - K5220 | Cow milk allergy | 2,159 (0·2%) | 4·1 ± 11·2 | 681 ± 2,129 | 2 (0·6%) | 622 |
| - A040-A044, B962 | *E. coli* | 1,817 (0·2%) | 4·6 ± 3·0 | 649 ± 720 | 1 (0·3%) | 480 |
| - A010-A014 | Typhoid and paratyphoid | 1,263 (0·1%) | 3·9 ± 2·4 | 455 ± 364 | 0 (0%) | 261 |
| - B770 | Ascariasis | 578 (0·1%) | 2·9 ± 2·4 | 383 ± 783 | 0 (0%) | 113 |
| - A00 | Cholera | 135 (0%) | 3·0 ± 2·3 | 488 ± 653 | 0 (0%) | 27 |
| - A047 | *Clostridioides difficile* enterocolitis | 131 (0%) | 9·3 ± 11·9 | 2,019 ± 3,982 | 4 (1·2%) | 397 |

*1 $PPP = 12·15 baht; ICD-10, International Classification of Disease Codes, 10^th^ Edition; LOS, length of stay; SD, standard deviation; DALYs, disability-adjusted life years.

**Table S4 Adjusted (aOR) odds ratio of risk factors among children under five years old admissions with diarrhoeal illness**

| Variable | aOR (95% CI) for high-cost admission | p-value | aOR (95% CI) for diarrhoeal-associated death | p-value |
| --- | --- | --- | --- | --- |
| **Sex** |  |  |  |  |
| - Female | Reference | – | Reference | – |
| - Male | 1·03 (1·00–1·06)* | 0·021 | 0·85 (0·66–1·09) * | 0·20 |
| **Hospital level** |  |  |  |  |
| - Primary | Reference | – | Reference | – |
| - Secondary | 1·57 (1·52–1·62) * | <0·0001 | 1·90 (1·33–2·71) * | 0·0004 |
| - Tertiary | 1·79 (1·72–1·85) * | <0·0001 | 1·93 (1·32–2·83) * | 0·0008 |
| **Region** |  |  |  |  |
| - Southern | Reference | – | Reference | – |
| - Northeastern | 0·98 (0·94–1·03) | 0·46 | 0·72 (0·50–1·03) | 0·07 |
| - Central | 3·04 (2·92–3·17) * | <0·0001 | 1·20 (0·84–1·71) | 0·32 |
| - Northern | 1·78 (1·69–1·87) * | <0·0001 | 0·63 (0·36–1·10) | 0·10 |
| - Eastern | 0·93 (0·87–0·99) * | 0·023 | 1·24 (0·75–2·06) | 0·40 |
| - Western | 1·12 (1·05–1·20) * | 0·0008 | 1·39 (0·83–2·33) | 0·21 |
|  |  |  |  |  |
| **Insurance scheme** |  |  |  |  |
| - UCS | Reference | – | Reference | – |
| - CSMBS/Officer | 1·58 (1·49–1·68) * | <0·0001 | 0·23 (0·03–1·67) | 0·15 |
| **Pathogen** |  |  |  |  |
| - Non-specific | Reference | – | Reference | – |
| - Typhoid and paratyphoid | 0·99 (0·77–1·29) | 0·96 | – | – |
| - *Salmonella* | 1·34 (1·23–1·46) * | <0·0001 | 1·31 (0·71–2·43) | 0·39 |
| - Amoeba | 0·92 (0·77–1·11) | 0·39 | 2·48 (0·35–17·83) | 0·37 |
| - Rotavirus | 3·37 (3·11–3·66) * | <0·0001 | 0·63 (0·22–1·85) | 0·40 |
| - *Strongyloides* | 2·14 (0·63–7·26) | 0·22 | – | – |
| - *E. coli* | 1·34 (1·14–1·57) * | 0·0003 | 0·29 (0·03–2·52) | 0·26 |
| - Cow milk allergy | 1·54 (1·28–1·85) * | <0·0001 | 2·69 (0·60–12·06) | 0·19 |
| - *C. difficile* | 1·66 (0·98–2·83) | 0·06 | 8·82 (1·55–50·31) * | 0·014 |
| - Ascariasis | 1·27 (0·78–2·07) | 0·34 | – | – |
| **Comorbidities** |  |  |  |  |
| - COVID-19 | 8·24 (7·44–9·12) * | <0·0001 | 4·98 (1·20–20·58) * | 0·027 |
| **Complications** |  |  |  |  |
| - Sepsis | 2·28 (1·99–2·62) * | <0·0001 | 7·39 (4·45–12·28) * | <0·0001 |
| - Dialysis | – | – | 209·21 (17·26–2536·33) * | <0·0001 |
| - Respiratory failure | 254·46 (190·96–339·08) * | <0·0001 | 2911·79 (2095·85–4045·39) * | <0·0001 |
| **Other factors** |  |  |  |  |
| - Readmissions | 0·88 (0·82–0·94) * | 0·0002 | 1·64 (0·81–3·31) | 0·17 |
| - OSI (per unit) | 1·02 (1·02–1·02) * | <0·0001 | 1·00 (0·99–1·01) | 0·58 |
| - Length of stay (days) | 2·20 (2·19–2·22) * | <0·0001 | 0·88 (0·85–0·90) * | <0·0001 |

* p-value < 0·05, UCS, Universal Coverage Scheme; CSMBS, Civil Servant Medical Benefits Scheme; DM, diabetes mellitus; HT, hypertension; COVID, coronavirus disease; CKD, chronic kidney disease; COPD, chronic obstructive pulmonary disease; CVD, cardiovascular disease; CHF, congestive heart failure; OSI, Oxford Stringency Index.

**
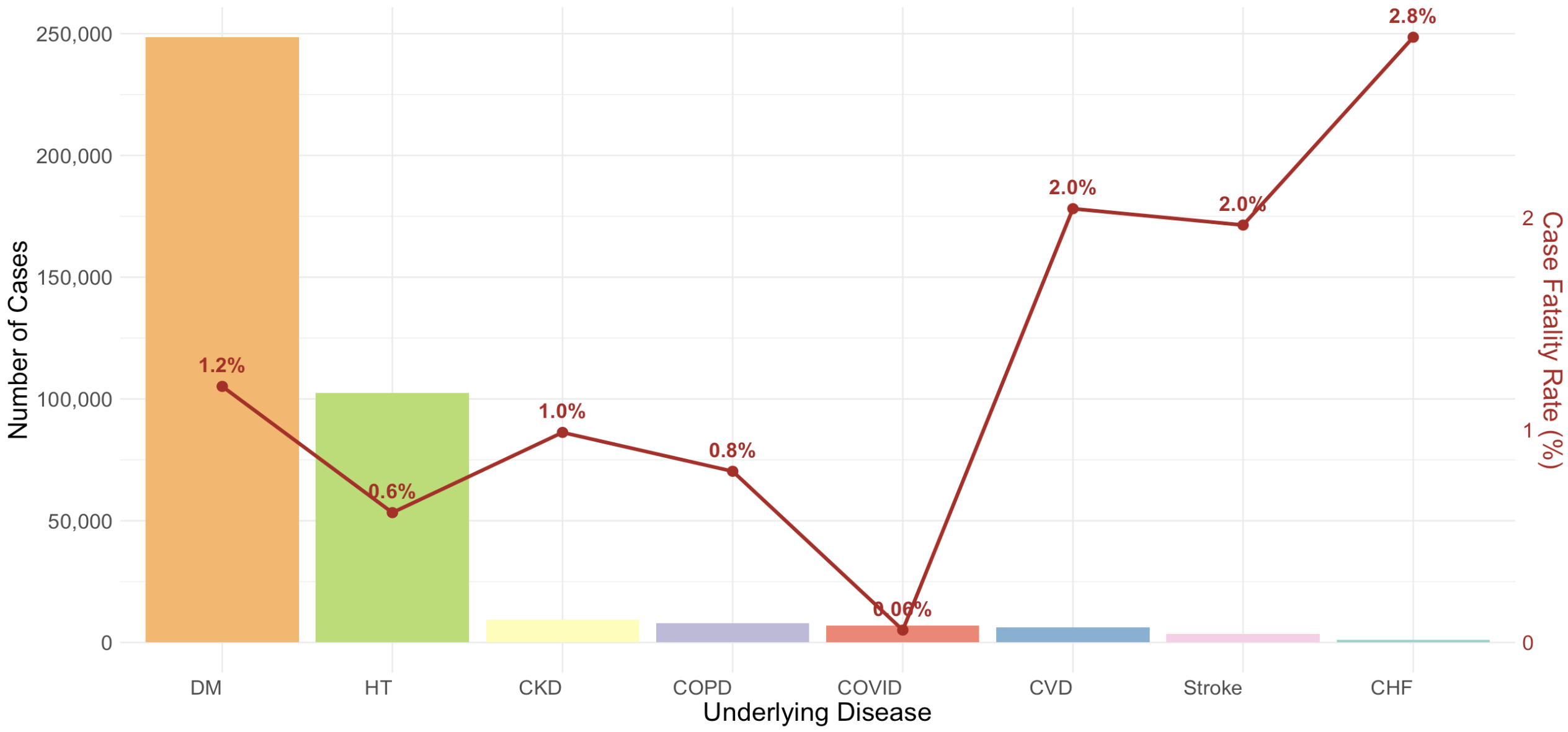
**

**Supplementary Figure S5 Number of diarrhoeal admissions and corresponding case fatality rates by underlying comorbidity.**

The coloured bars represent the total number of hospital admissions for patients presenting with each specified underlying disease (left axis). The solid red line denotes the corresponding case fatality rate (CFR) expressed as a percentage for each comorbidity group (right axis).

DM, diabetes mellitus; HT, hypertension; COVID, coronavirus disease; CKD, chronic kidney disease; COPD, chronic obstructive pulmonary disease; CVD, cardiovascular disease; CHF, congestive heart failure.

**
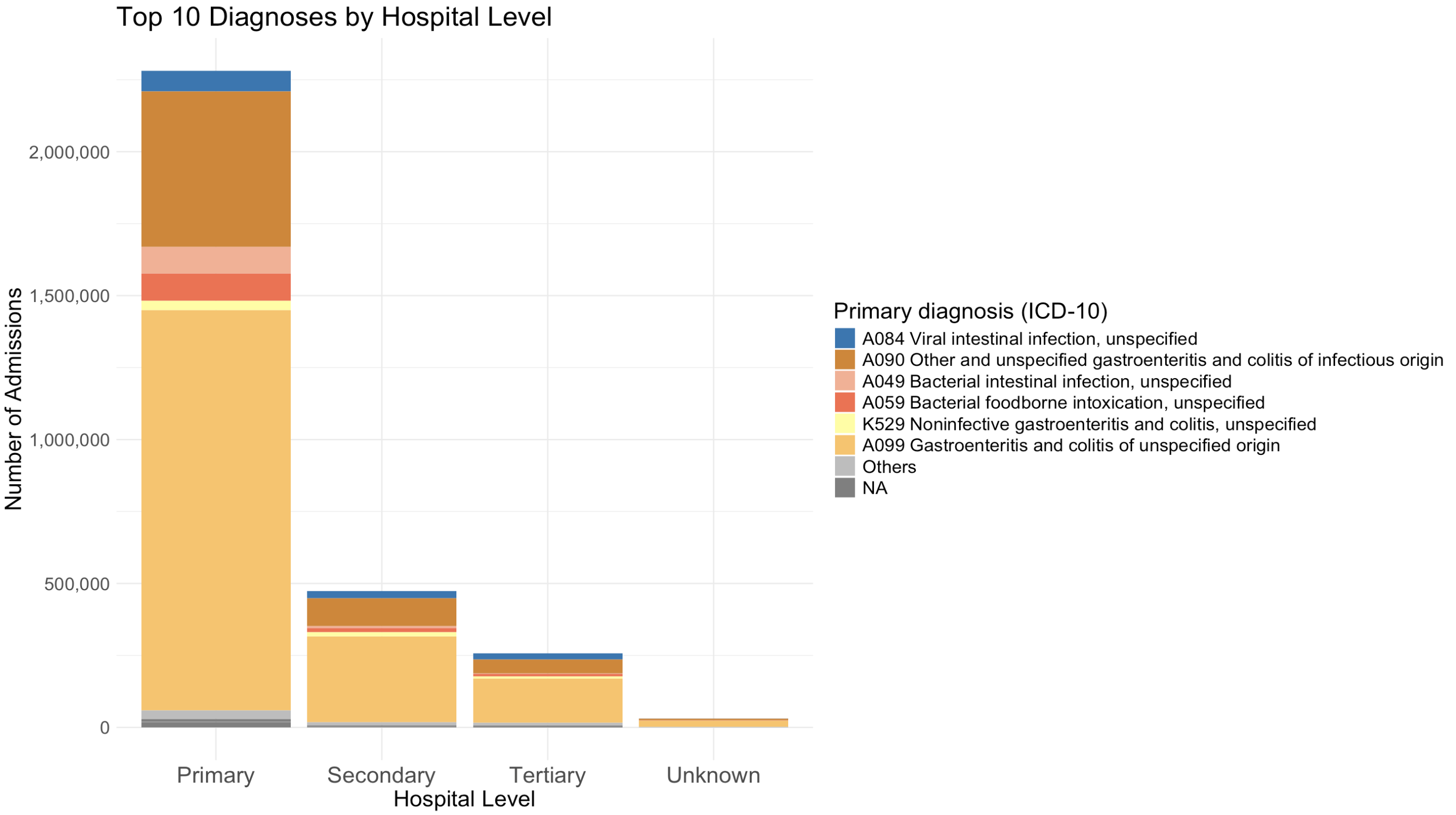
**

**Supplementary Figure S6 Distribution of the most common primary diarrhoeal diagnoses by hospital level in Thailand.**

Stacked bars show the number of admissions across primary (< 150 beds, community hospitals), secondary (151–499 beds, general hospitals), tertiary (> 500 beds, regional hospitals), and unknown hospital levels, with coloured segments representing ICD-10 primary diagnosis categories. Across all hospital levels, admissions were dominated by non-specific diarrhoeal diagnoses, particularly A099 (gastroenteritis and colitis of unspecified origin) and A090 (other and unspecified gastroenteritis and colitis of infectious origin), whereas pathogen-specific diagnoses accounted for a smaller proportion of admissions.

**
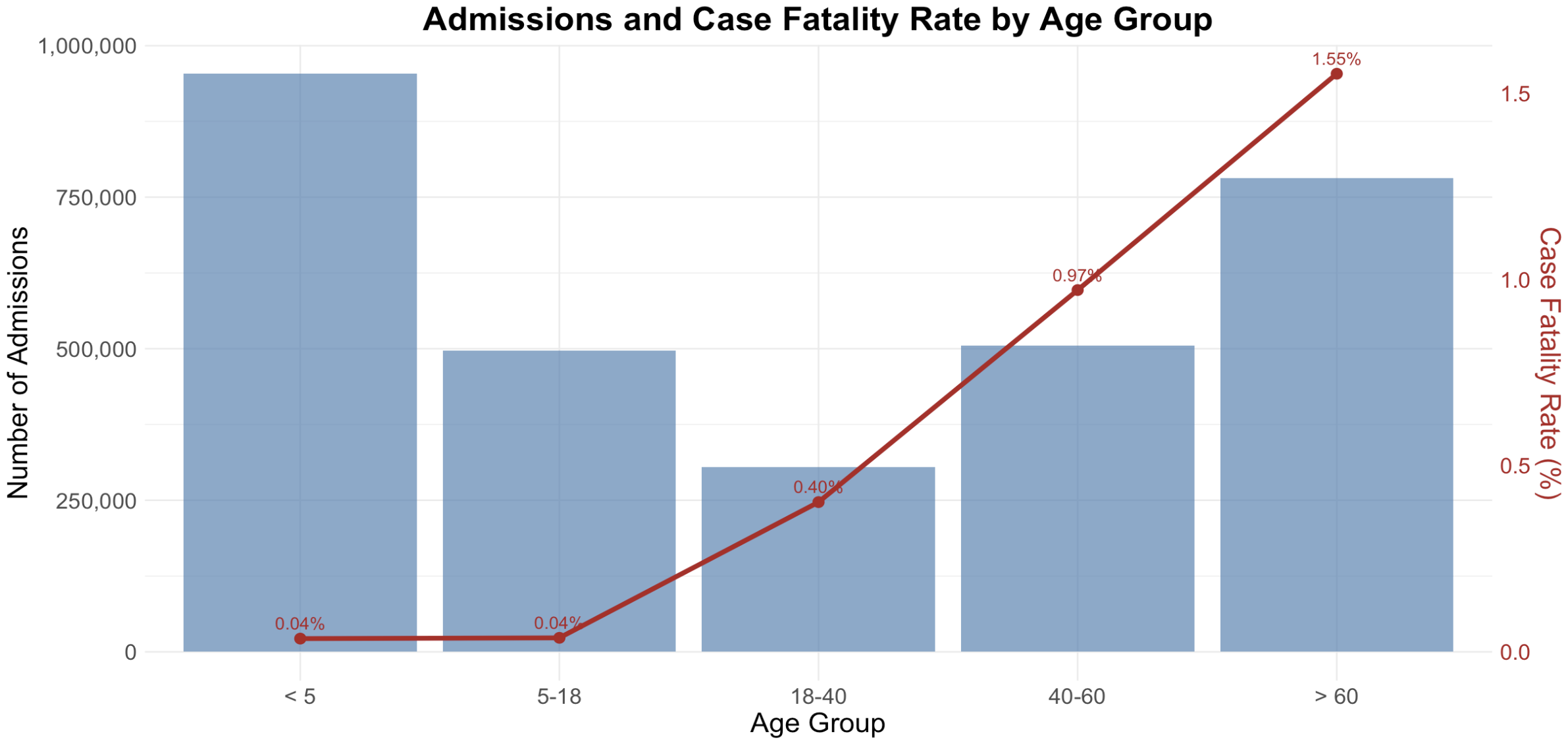
**

**Supplementary Figure S7 Number of admissions and case fatality rate by age group among hospitalised patients with diarrhoeal disease in Thailand.**

Bars show the number of admissions in each age group, and the red line shows the corresponding case fatality rate (%). Admissions were highest among children aged younger than 5 years and adults aged older than 60 years, whereas case fatality increased progressively with age and was highest among older adults.

**
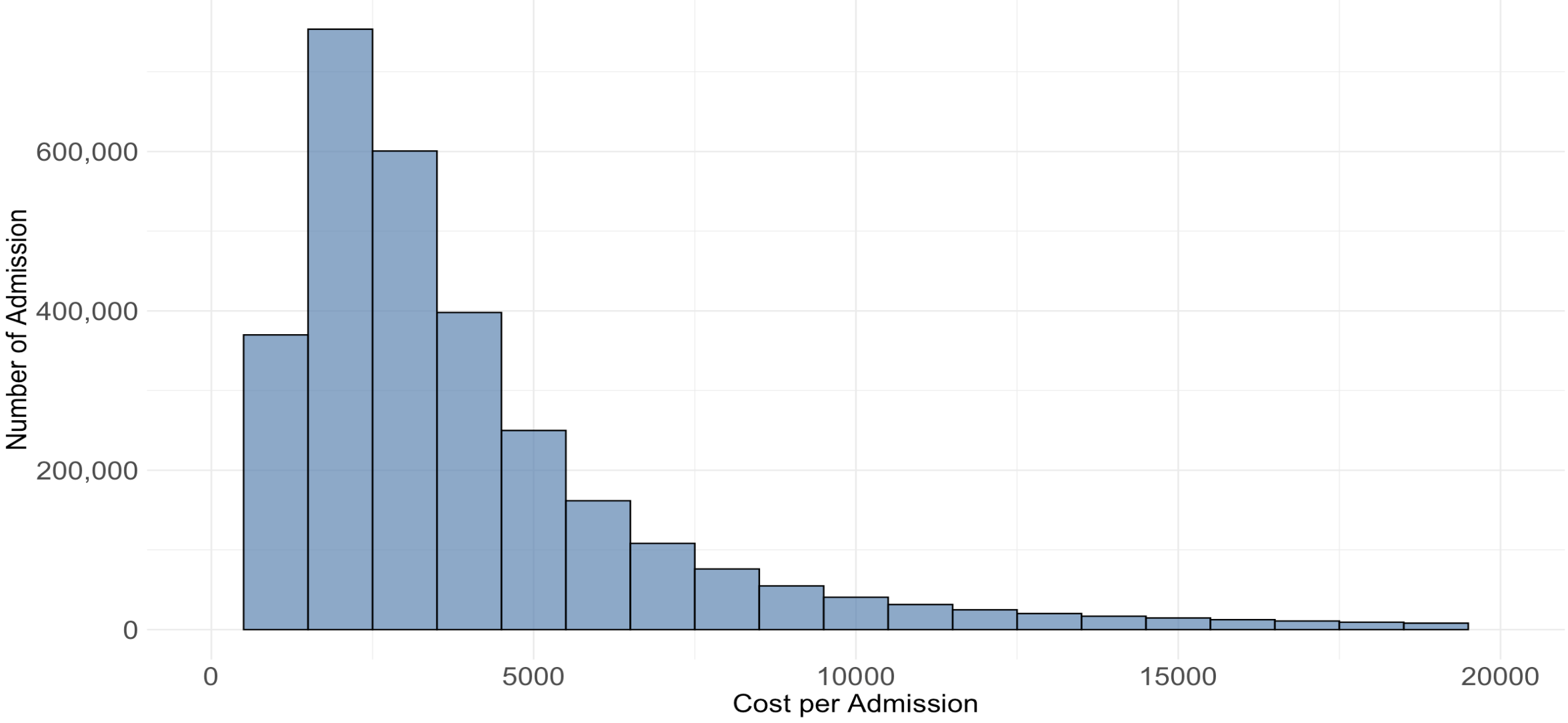
**

**Supplementary Figure S8 Frequency distribution of cost per diarrhoeal admission (up to THB 20,000).**

This histogram illustrates the volume of hospital admissions (y-axis) distributed across different expenditure intervals in Thai Baht (THB) (x-axis). The x-axis is capped at THB 20,000 to clearly visualise the primary distribution of hospital costs. The strongly right-skewed shape demonstrates that the vast majority of diarrhoeal episodes incur relatively low healthcare costs, peaking sharply in the lower expenditure brackets, followed by a long tail representing a progressively smaller proportion of high-cost hospitalisations.

**
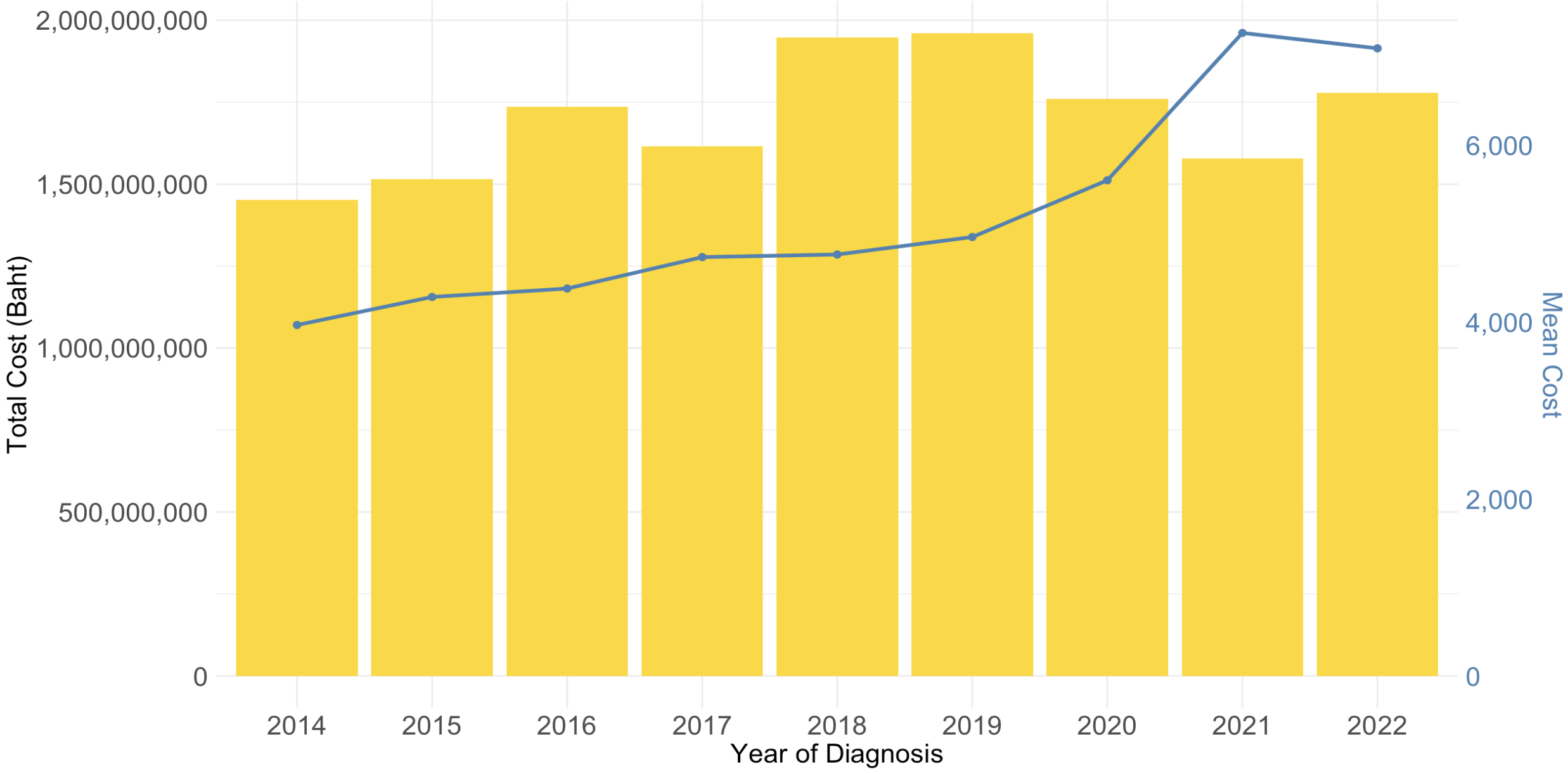
**

**Supplementary Figure S9 Annual total hospital cost and mean cost per admission among patients admitted with diarrhoeal disease in Thailand,
2014–2022.**

Yellow bars show the total annual hospital cost, and the blue line shows the mean cost per admission. Total costs remained substantial throughout the study period, while mean cost per admission increased over time, with a marked rise in the pandemic period (2020-2022).

**
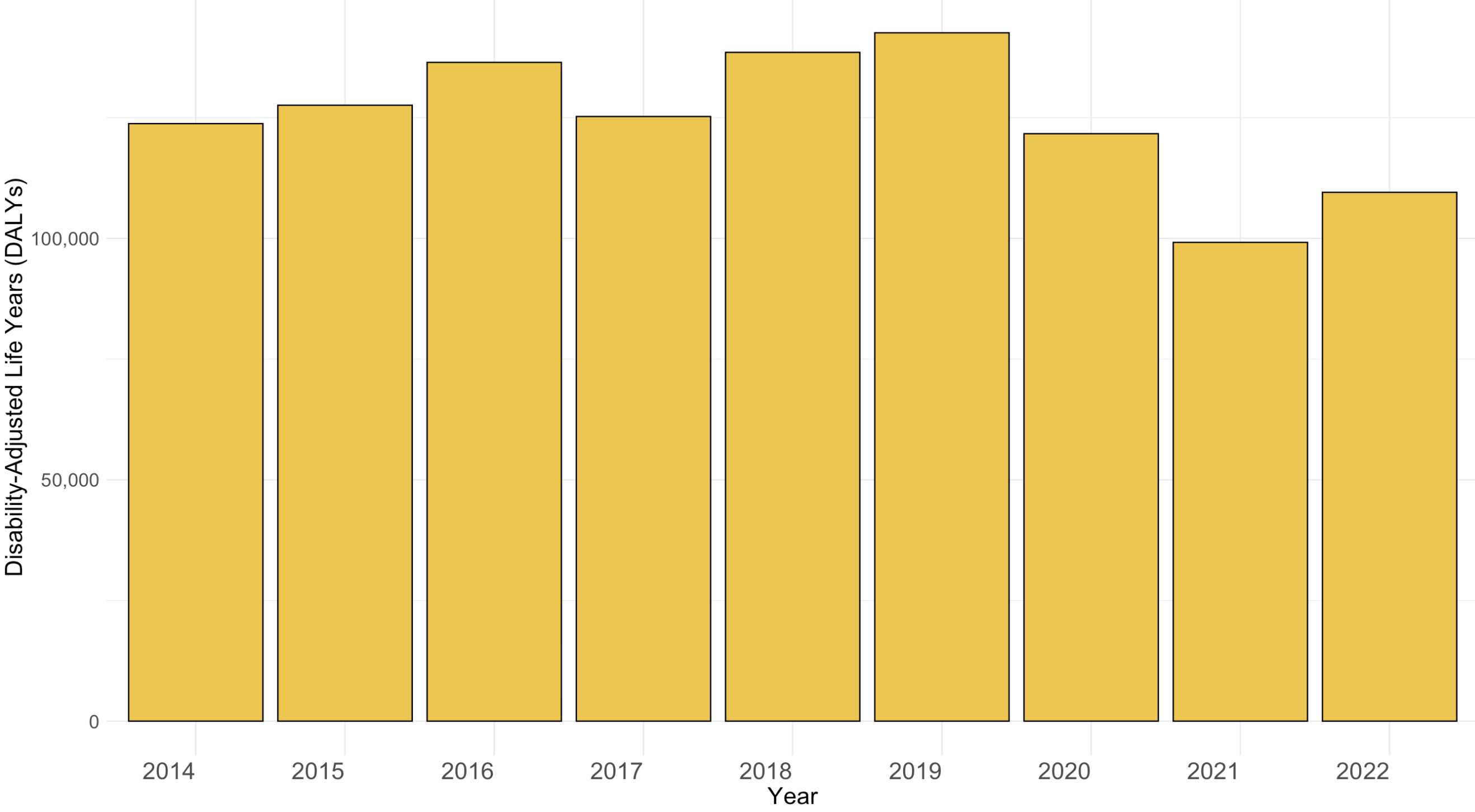
**

**Supplementary Figure S10 Annual trends in total Disability-Adjusted Life Years (DALYs) attributed to diarrhoeal admissions (2014–2022).**

This bar chart illustrates the total number of DALYs (y-axis) recorded each year (x-axis) across the study period. The data shows relatively stable but fluctuating disease burden during the pre-pandemic years (2014–2019), followed by a notable decrease in total DALYs during the initial COVID-19 pandemic period (2020–2021), likely reflecting reduced overall hospital admission volumes.

**
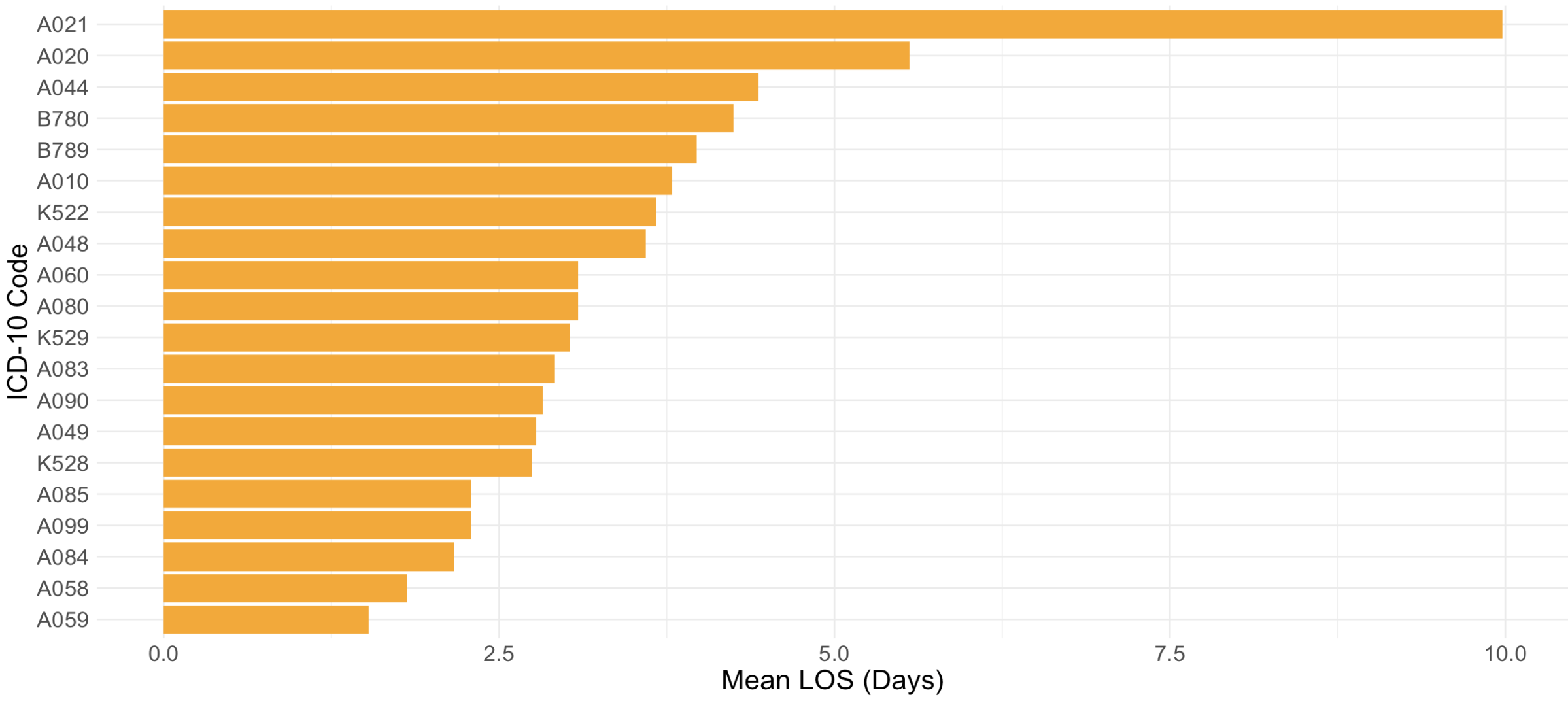
**

**Supplementary Figure S11 Top 20 primary diagnoses by mean length of stay (LOS)**

This horizontal bar chart ranks the 20 diarrhoeal diagnoses with the highest average hospital duration. The y-axis lists the specific ICD-10 diagnostic codes, while the x-axis displays the mean length of stay in days. The distribution highlights that severe and systemic bacterial infections, particularly *Salmonella*, are associated with significantly longer hospitalisation periods compared to unspecified viral infections and foodborne intoxications.

**ICD-10 Code Definitions:**

- **A02.1:** *Salmonella* sepsis
- **A02.0:** *Salmonella* enteritis
- **A04.4:** Other intestinal *Escherichia coli* infections
- **B78.0:** Intestinal strongyloidiasis
- **B78.9:** Strongyloidiasis, unspecified
- **A01.0:** Typhoid fever
- **K52.2:** Allergic and dietetic gastroenteritis and colitis
- **A04.8:** Other specified bacterial intestinal infections
- **A06.0:** Acute amebic dysentery
- **A08.0:** Rotaviral enteritis
- **K52.9:** Noninfective gastroenteritis and colitis, unspecified
- **A08.3:** Other viral enteritis
- **A09.0:** Other and unspecified gastroenteritis and colitis of infectious origin
- **A04.9:** Bacterial intestinal infection, unspecified
- **K52.8:** Other specified noninfective gastroenteritis and colitis
- **A08.5:** Other specified viral intestinal infections
- **A09.9:** Gastroenteritis and colitis of unspecified origin
- **A08.4:** Viral intestinal infection, unspecified
- **A05.8:** Other specified bacterial foodborne intoxications
- **A05.9:** Bacterial foodborne intoxication, unspecified
